# Skp2 modulates proliferation, senescence and tumorigenesis of glioma

**DOI:** 10.1186/s12935-020-1144-z

**Published:** 2020-03-06

**Authors:** Juan Wu, Hong-kai Su, Zhi-hui Yu, Shao-yan Xi, Cheng-cheng Guo, Zhe-yu Hu, Yue Qu, Hai-ping Cai, Yi-ying Zhao, Hua-fu Zhao, Fu-rong Chen, Yu-fan Huang, Shing-shun Tony To, Bing-hong Feng, Ke Sai, Zhong-ping Chen, Jing Wang

**Affiliations:** 1 Guangzhou Key Laboratory of Translational Medicine on Malignant Tumor Treatment, Affiliated Tumor Hospital of Guangzhou Medical University, Guangzhou, 510060 Guangdong People’s Republic of China; 2 Department of Neurosurgery/Neuro-oncology, Sun Yat-sen University Cancer Center, State Key Laboratory of Oncology in South China, Collaborative Innovation Center for Cancer Medicine, Guangzhou, 510060 Guangdong People’s Republic of China; 3 Department of Breast Cancer Medical Oncology, Hunan Cancer Hospital, Changsha, 410013 People’s Republic of China; 4 Department of Pharmacology, College of Pharmacy, Guangdong Pharmaceutical University, Guangzhou, 510006 Guangdong People’s Republic of China; 5 Institute of Translational Medicine, Department of Neurosurgery and Shenzhen Key Laboratory of Neurosurgery, Shenzhen Second People’s Hospital, The First Affiliated Hospital of Shenzhen University, Shenzhen, 518035 Guangdong People’s Republic of China; 6 Department of Health Technology and Informatics, The Hong Kong Polytechnic University, Hung Hom, Hong Kong, People’s Republic of China

**Keywords:** Glioma, S-phase kinase-associated protein 2 (Skp2), Glioma stem-like cells, Lovastatin, SZL-P1-41

## Abstract

**Background:**

Gliomas represent the largest class of primary central nervous system neoplasms, many subtypes of which exhibit poor prognoses. Surgery followed by radiotherapy and chemotherapy has been used as a standard strategy but yielded unsatisfactory improvements in patient survival outcomes. The S-phase kinase protein 2 (Skp2), a critical component of the E3-ligase SCF complex, has been documented in tumorigenesis in various cancer types but its role in glioma has yet to be fully clarified. In this study, we investigated the function of Skp2 in the proliferation, stem cell maintenance, and drug sensitivity to temozolomide (TMZ) of glioma.

**Methods:**

To investigate the role of Skp2 in the prognosis of patients with glioma, we first analyzed data in databases TCGA and GTEx. To further clarify the effect of Skp2 on glioma cell proliferation, we suppressed its level in glioblastoma (GBM) cell lines through knockdown and small molecule inhibitors (lovastatin and SZL-P1-41). We then detected cell growth, colony formation, sphere formation, drug sensitivity, and in vivo tumor formation in xenograft mice model.

**Results:**

Skp2 mRNA level was higher in both low-grade glioma and GBM than normal brain tissues. The knockdown of Skp2 increased cell sensitivity to TMZ, decreased cell proliferation and tumorigenesis. In addition, Skp2 level was found increased upon stem cells enriching, while the knockdown of Skp2 led to reduced sphere numbers. Downregulation of Skp2 also induced senescence. Repurposing of lovastatin and novel compound SZL-P1-41 suppressed Skp2 effectively, and enhanced glioma cell sensitivity to TMZ in vitro and in vivo.

**Conclusion:**

Our data demonstrated that Skp2 modulated glioma cell proliferation in vitro and in vivo, stem cell maintenance, and cell sensitivity to TMZ, which indicated that Skp2 could be a potential target for long-term treatment.

## Background

Gliomas represent the largest class of primary central nervous system neoplasms, many subtypes of which exhibit poor prognoses. Even with aggressive treatment strategies, i.e. surgery followed by irradiation and chemotherapy, the prognoses of diffuse glioma patients remained unsatisfactory [1]. Encouraging results were found with temozolomide (TMZ) which provided certain extents of survival benefits. However, resistance to TMZ has been a cumbersome obstacle hindering treatment outcomes, for which alternative therapeutics are still limited. As such, efficient ways to improve the long-term prognosis of patients, such as overcoming TMZ resistance, are urgently required.

To this end, the S-phase kinase-associated protein 2 (Skp2) has been identified as a potential prospect worth investigating. It is a member of the F-box protein family and forms the Skp-Cullin-F-box complex (SCF complex) with Skp1, Cullin-1, and Rbx1. The SCF complex has been showed to trigger the ubiquitination and degradation of downstream proteins, and Skp2 recognizes substrates for proteasome degradation [2]. Skp2 targets cell cycle progression through the ubiquitin-mediated degradation of G1 checkpoint CDK inhibitors, p21^Cip1/Waf1^ and p27^Kip1^ [3, 4]. p21^Cip1/Waf1^ is a broad-acting cyclin-dependent kinase inhibitor, and its stability is essential for proper cell cycle progression and cell fate decisions. An increase in p27^Kip1^ has been found to significantly reduce Mixed-Lineage Leukemia self-renewal ability, promote the monocytic differentiation of leukemic blasts, and induce cell death [5]. Skp2 has shown important roles in the development and progression of tumors as it was found to be frequently overexpressed in various human malignancies like hepatocellular carcinoma, breast cancer, lung cancers, prostate cancer, and osteosarcoma [2, 6–11]. In hepatocellular carcinoma, Skp2 was identified at a significantly increased level compared with paired normal tissue, and was correlated with tumor grade, size, and metastases. The mRNA level of Skp2 was also found increased in triple-negative breast cancer (TNBC) and was significantly associated with poor prognoses, and its suppression in TNBC inhibited cell proliferation and G1/S transition; evincing Skp2 as a protooncoprotein.

Although it was reported that Skp2 could promote cell growth, migration, and invasion in glioma cells, the detailed function of Skp2 remains unclear [12]. In this study, we analyzed data from database to elucidate the role of Skp2 in clinical progression of glioma. We then modulated Skp2 level by knockdown or small molecule inhibition, and then examined the functions of Skp2 in the development of glioma through in vitro and in vivo assays. Our results indicated that Skp2 could serve as a potential target to improve the prognosis of diffuse glioma patients and that lovastatin and SZL-P1-41might be candidate medicines.

## Methods

### Cell lines

Cell lines U87, U118, U373, U343 and 293T were maintained from the State Key Laboratory of Oncology in South China. A172, U138, LNZ308 and normal astrocyte cell line (Ast) were obtained from Dr. Shing-shun Tony To, Department of Health Technology and Informatics, The Hong Kong Polytechnic University. The WHO classification and IDH mutation status of glioma cell lines applied in our study was listed in Additional file 1: Table S1. All glioma cells lines were maintained in DMEM (Thermo Fisher Scientific, Waltham, MA, USA) containing fetal bovine serum (FBS, Genetimes Technology Inc, Shanghai, China) at 37 °C in a humidified incubator with 5% CO_2_. The astrocytes were maintained in Astrocyte Medium (ScienCell Research Laboratories, San Diego, CA, USA) supplemented with FBS, astrocyte growth supplement and penicillin/streptomycin (Thermo Fisher Scientific, Waltham, MA, USA). The glioma stem-like cells were cultured in conditional medium (DMEM/F12, Thermo Scientific) supplemented with B27 (Thermo Fisher Scientific), epidermal growth factor (EGF) (20 ng/ml, Peprotech, Rocky Hill, NJ, USA), basic fibroblast growth factor (bFGF) (20 ng/ml, Peprotech, Rocky Hill, NJ, USA), and 1% penicillin/streptomycin.

### Overall survival (OS) analysis based on database

We applied Gene Expression Profiling Interactive Analysis (GEPIA) to investigate the expression level of Skp2 in 24 tumor types and differential expression in GBM and low-grade glioma (LGG) compared with normal brain tissues. GEPIA is an interactive web application for gene expression analysis based on 9736 tumors and 8587 normal samples from the TCGA and the GTEx databases, using the output of a standard processing pipeline for RNA sequencing data [13]. GEPIA is a timesaving and intuitive tool for making full use of big genomic data in TCGA and GTEx (http://gepia.cancer-pku.cn/index.html) [14]. We also downloaded the clinical data of GBM and LGG from TCGA and analyzed the OS of the cohort with different Skp2 expression levels. The clinical information was summarized in Additional file 1: Table S2.

### Viral infection

The preparation of viral particles was performed as in our previous publication [15]. Briefly, supernatants from 293T cells containing viral particles were collected 48 h after transfection and then applied to the target cells. The virus infection was repeated 24 h later by freshly collected virus supernatants. The cells were selected by puromycin (Thermo Fisher Scientific) at a final concentration of 1.5 μg/ml for continuous 2 weeks 3 days after the infection.

### Western blot analysis

Western blot analyses were performed as described previously [16, 17]. The antibodies used were as follows: mouse anti-Skp2p45 (Thermo Fisher Scientific), anti-p21^Cip1/Waf1^ (Proteintech, Rosemont, IL, USA), anti-β-catenin (Abcam, Cambridge, MA, USA), β-tubulin (Proteintech), and β-actin (CST, Danvers, MA, USA), and rabbit anti-p27^Kip1^ (Affinity, Cincinnati, OH, USA). The images were captured for further analysis on a BIO-RAD ChemiDoc Imaging System (BIO-RAD, Louisville, KY, USA).

### Cell cycle analysis

Cells were harvested, fixed with 70% ethanol overnight at 4 °C, and stained with propidium iodide (KeyGEN, Jiangsu, China) in the dark for 1 h. Cell suspensions were subjected to flow cytometry with an ACEA NovoCyte system (ACEA Biosciences, Inc., Santa Clara, CA). Cell cycle distribution was analyzed with 100,000 events for each sample.

### Flow cytometry analysis

Flow cytometry analysis was used to detected apoptotic cell death. Briefly, cell  suspensions were blocked with 10% BSA for 10 min. Apoptosis were detected using Annexin V–FITC/PI detection kit (BD biosciences, New Jersey, USA). Flow cytometry analysis was performed using a CytoFLEX (Beckman Coulter Inc., CA, USA) flow cytometer equipped with CytExpert software with 20,000 events recorded for each sample.

### Cell proliferation detection

Cells were quantified and seeded into 96-well plates. Cell viability was detected with a CCK8 kit (Yeasen, Shanghai, China) for 6 continuously days. Growth curves were then made according to the observation density (OD) values.

### Drug sensitivity test

Cells viability was tested using the CCK8 assay after treatment. Lovastatin (C_24_H_36_O_5_, 404.54 g/mol, Merck Millipore, Darmstadt, Hessen, Germany) and SZL-P1-41 (C_24_H_24_N_2_O_3_S, 420.52 g/mol, Bio-Techne, Minneapolis, MN, USA) were used for combination treatment with TMZ (Selleck,Houston, TX, USA). Lovastatin (25 mg) was dissolved in DMSO (6.18 ml) to get the stock solution (10 mmol/l), which was further diluted into 1 μmol/l with culture medium for further application. Similarly, the stock solution of SZL P1-41 (5 mmol/l) was prepared by dissolving 10 mg in 4.756 ml of DMSO, which was further diluted into 1 mmol/l with culture medium for further use. Cells were counted and seeded into 96-well plates on day 1, and pretreated with lovastatin or SZL-P1-41 on day 2. The final concentration of lovastatin and SZL P1-41 applied to cells were 10 nmol/l and 20 μmol/l respectively. TMZ was applied on day 3 for 72 h (together with lovastatin or SZL-P1-41) and the cell viability was detected. The survival rate was drawn according to the OD and compared to nontreated cells. The half-maximal inhibitory concentration (IC50) was calculated by GraphPad Prism 5 (GraphPad Software, San Diego, CA, USA). All experiments were repeated at least three times, and each concentration had four independent repeats.

### Colony and sphere formation assay

Cells were trypsinized, resuspended in single-cell supernatant, seeded into 6-well plates for colony formation assay in common medium, or into an ultralow attachment 24-well plate (Corning, NY, USA) for sphere formation assay in stem-like cell conditional medium, at a density of 1000 cells per well as reported [16]. After culturing for 10–15 days, the colonies were fixed with 4% paraformaldehyde and stained with crystal violet, colonies with over 50 cells were counted. The spheres were counted after being cultured for 10 to 14 days.

### Senescence staining

The assay was performed as reported previously [16]. Briefly, cells were plated into 6-well plates and cultured for 3 to 4 days, and stained with a Senescence Activated β-galactosidase (SA-βGal) Staining Kit (Beyotime, Guangzhou, China) following the manufacturer’s instructions. Those green cells were observed and counted under a microscope.

### Xenograft transplantation in vivo

The in vivo animal work followed previously published protocol [18]. Four-week-old female BALB/C nude mice (Charles River Laboratories, Beijing, China) were raised in Sun Yat-sen University Cancer Center (SYSUCC) animal facilities. Single-cell suspension containing 1 × 10^7^ cells was subcutaneously injected into the back of nude mice. The resulting tumor size was measured with a Vernier caliper every 3 days and tumor weight was determined immediately after separated from mice. Tumor-bearing mice were treated with TMZ when tumor volume reached 80 mm^3^. The mice were observed for over 4 weeks and sacrificed before dying of tumor burden.

### Statistical analysis

Data obtained from both in vitro and in vivo are expressed as the mean ± standard error. p < 0.05 was considered statistically significant. Statistical analysis was performed with Graph Pad Prism 5.0.

## Results

### High expression level of Skp2 predicted a poor prognosis in glioma

**Fig. 1:**
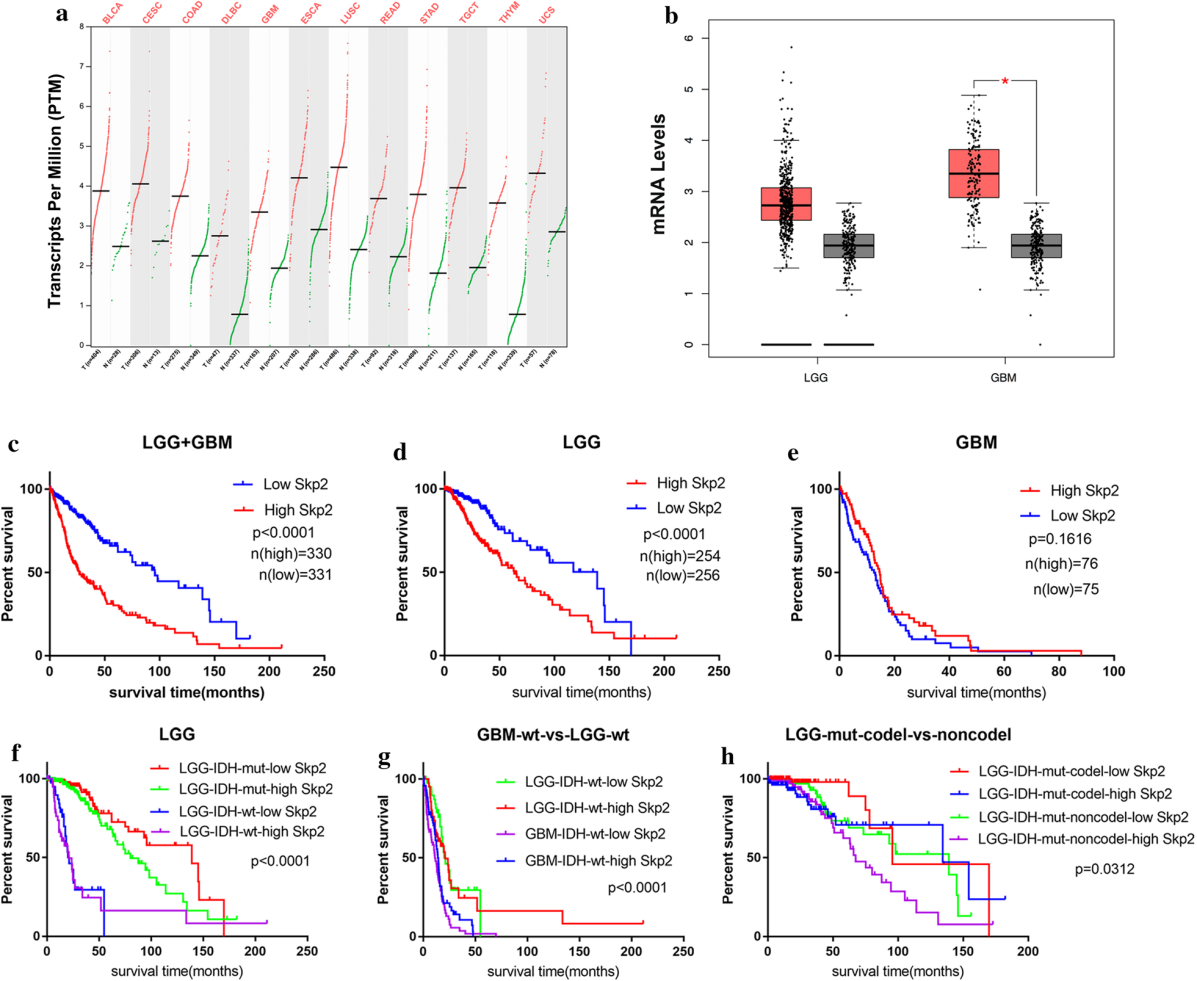
High expression level of Skp2 predicted poor prognosis in glioma. **a** Skp2 was overexpressed in various types of tumor tissues compared with paired normal tissues. **b** Skp2 mRNA levels were high in both LGG and GBM, but only significant in GBM. **c** In glioma (including both LGG and GBM), the OS was shorter for patients with high-level of Skp2 (n = 330) than those with low-level of Skp2(n = 331) (p < 0.0001). **d** In LGG, the OS was poorer in patients with high-level of Skp2 (n = 254) than those with a low-level of Skp2 (n = 256) (p < 0.0001). **e** There was no significant difference on OS between different mRNA levels of Skp2 in GBM. **f** The OS was analyzed according the IDH mutation status and Skp2 expression level (n = 507). **g** The OS was analyzed in LGG (n = 94) and GBM (n = 137) patients with IDH^wt^ and different Skp2 levels. **h** The OS was analyzed by status of 1p19q co-deletion and Skp2 expression level in IDH^mut^ LGG patients (n = 413) (BLCA: Bladder Urothelial Carcinoma; CESC: Cervical squamous cell carcinoma and endocervical adenocarcinoma; COAD: Colon adenocarcinoma; DLBC: Lymphoid Neoplasm Diffuse Large B-cell Lymphoma; GBM: Glioblastoma; ESCA: Esophageal carcinoma; LUSC: Lung squamous cell carcinoma; READ: Rectum adenocarcinoma; STAD: Stomach adenocarcinoma; TGCT: Testicular Germ Cell Tumors; THYM: Thymoma; UCS: Uterine Carcinosarcoma)

Based on the RNA sequencing data from TCGA and GTEx, the web-based tool GEPIA, helped us analyzing the expression of the genes [13]. Skp2 was significantly over expressed in various types of tumor tissues (Fig. 1a). Its expression in LGG and GBM was all elevated compared to normal tissues, especially in GBM (LGG, n = 518; GBM, n = 162; normal brain, n = 207, Fig. 1b). The OS of diffuse glioma patients with low-level of Skp2 was greater than those patients with high expression level (n = 661, p < 0.0001, Fig. 1c). Further analyses showed that low expression of Skp2 led to better survival in LGG (n = 510, p < 0.0001, Fig. 1d) but not in GBM (n = 151, p = 0.1616, Fig. 1e). IDH1 mutation was a favorable factor in the prognosis of diffuse glioma especially in LGG based on the 2016 WHO classification [19]. We then dissected cohort of LGG into IDH1 mutant (mut) and wild type (wt), and analyzed patient OS. The patients’ prognoses were better with IDH1^mut^ and Skp2^low^ (Fig. 1f, p < 0.0001). We did not analyze the role of IDH1^mut^ in GBM since there were only 10 patients accompanied by IDH1^mut^. Although there were significant difference between the prognoses of patients with IDH1^wt^ LGG and IDH1^wt^ GBM, it might mainly due to the disease grading (Fig. 1g, p < 0.0001). Skp2 did not affect OS in IDH1^wt^ GBM, IDH^wt^ LGG and even IDH^mut^ LGG alone (Additional file 2: Figure S1a–c, p = 0.133, 0.6508, and 0.0613, respectively). Furthermore, 1p19q co-deletion was another favorable parameter of LGG prognosis [19]. We analyzed the role of 1p19q co-deletion in IDH^mut^ LGG (n = 413) patients since there was no 1p19q co-deletion in the IDH^wt^ LGG patients (n = 94). We found that the combination of 1p19q non-co-deletion and Skp2^high^ contributed to the poor prognoses in IDH^mut^ LGG compared with other three groups, 1p19q co-deletion/Skp2^low^, 1p19q co-deletion/Skp2^high^ and 1p19q non-co-deletion/Skp2^low^ (Fig. 1h, p = 0.0312). Skp2 level did not affect the prognoses of IDH^mut^ LGG patients with 1p19q co-deletion (Additional file 2: Figure S1d, p = 0.1267), but played a role in IDH^mut^ LGG patients without 1p19q co-deletion (Additional file 2: Figure S1e, p = 0.0433). Although the differential expression of Skp2 in GBM was more remarkable than in LGG (Fig. 1b), its role in prognosis was stronger in LGG, contributed in the earlier stages of IDH^mut^ LGG patients (Additional file 2: Figure S1c) and IDH^mut^ LGG patients without 1p19q co-deletion.

### Skp2 knockdown attenuated the growth of glioma cells both in vitro and in vivo

**Fig. 2:**
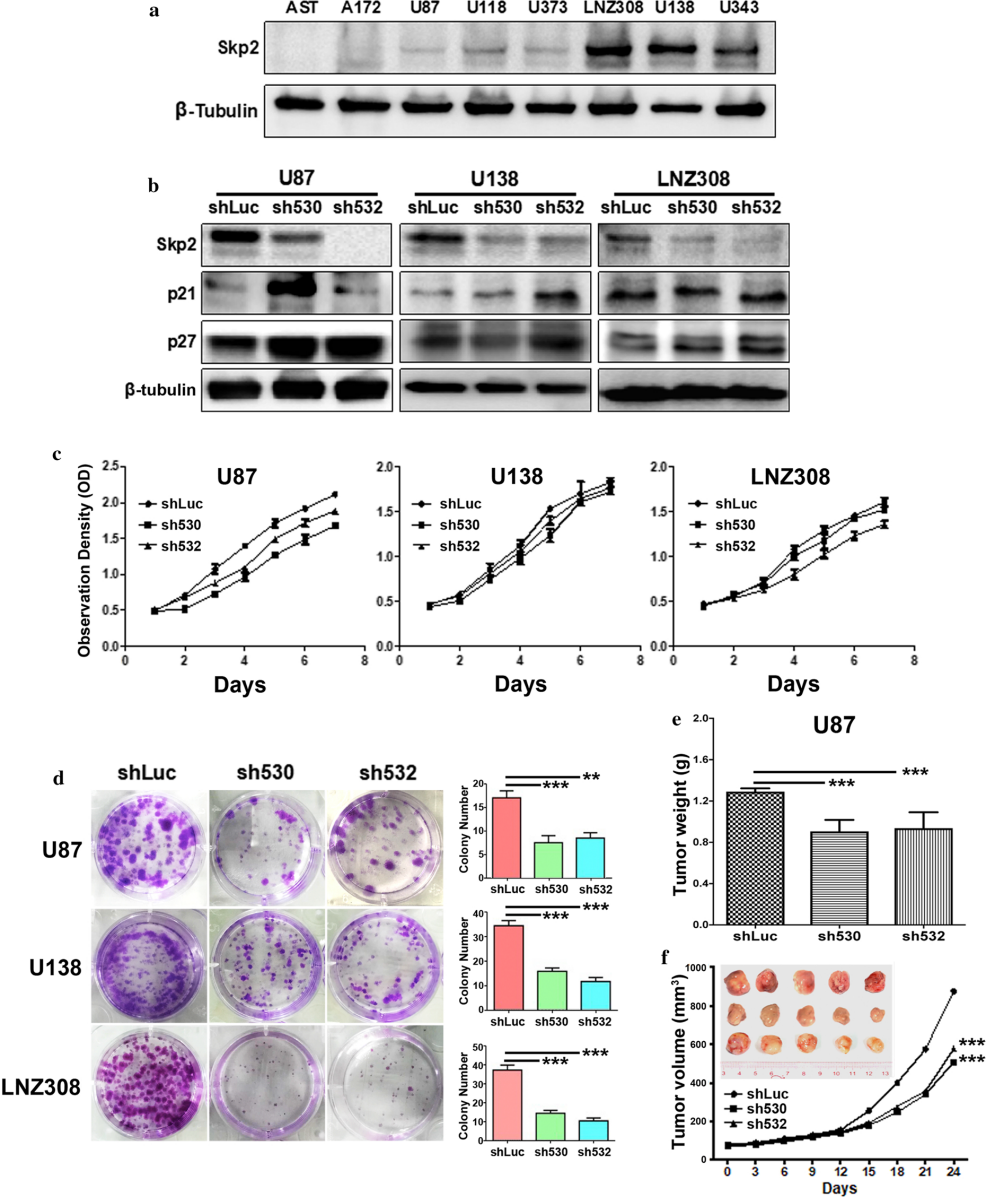
Downregulation of Skp2 attenuated in vitro and in vivo cell proliferation. **a** On Western blot analyses, the protein level of Skp2 in glioma cell lines (A172, U87, U118, U373, LNZ308, U138 and U343), as compared to normal astrocytes (AST), was enhanced. **b** Skp2 was successfully knocked-down by two shRNA fragments in U87, U138, and LNZ308 cells compared with the negative control shLuc. The protein levels of the downstream molecules p21^Cip1/Waf1^ and p27^Kip1^ were increased upon the knockdown of Skp2 in all three cell lines. **c** Cell proliferation was retarded slightly in U87, U138 and LNZ308 cells after the knockdown of Skp2. **d** The colony formation ability of the three cell lines was reduced after Skp2 knockdown. **e**, **f** The tumor weight and volume were smaller than the control group upon Skp2 knockdown in xenograft mouse models. **p < 0.01, ***p < 0.001

To determine the role of Skp2 in the proliferation of glioma cells, the expression level of Skp2 in 7 glioma cell lines (A172, U87, U118, U373, LNZ308, U138, and U343) was found stronger than in AST cells (Fig. 2a). Three cell lines with low, moderate, and strong level of Skp2 were chosen for Skp2 knockdown, namely U87, U138, and LNZ308. The knockdown was successful in all three cell lines which were confirmed by the enhancement of p21^Cip1/Waf1^ and p27^Kip1^ (Fig. 2b). Although the cell proliferation rates were attenuated slightly, the colony formation ability was significantly reduced after Skp2 knockdown (**p < 0.01, ***p < 0.001, Fig. 2c, d). In the xenograft mice model, both of the tumor size and weight were reduced upon Skp2 knockdown (***p < 0.001, Fig. 2e, f). Thus, Skp2 was important in glioma cell proliferation in vitro and in vivo.

### Knockdown of Skp2 increased the cell sensitivity to TMZ

**Fig. 3:**
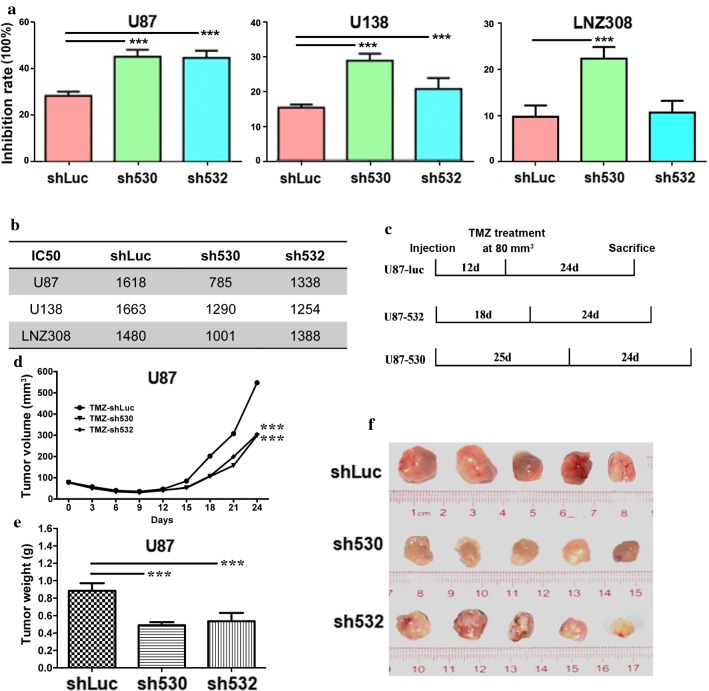
Downregulation of Skp2 sensitized glioma cells to TMZ. **a** After Skp2 knockdown, the cell sensitivity to TMZ (inhibition rate) was enhanced in all three cell lines (except sh532 of LNZ308 cells). **b** The IC50 of TMZ was reduced when Skp2 was knocked down in three cell lines. **c** The time course of tumor cell sensitivity to TMZ in vivo. **d** Tumor progression starting from the day TMZ was added. **e**, **f** Tumors and their corresponding weights after removal from the mice, sacrificed 24 days after TMZ treatment. ***p < 0.001

To test whether Skp2 affects the cell sensitivity to TMZ, the common clinical chemotherapeutic reagent for glioma, we treated Skp2-knockdown cells with TMZ. Upon Skp2 knockdown, the inhibition rate increased and the IC50 of TMZ decreased in all three cell lines (***p < 0.001, Fig. 3a, b). In xenograft tumor mice, TMZ was administrated when the tumor size reached 80 mm^3^ and the mice were then observed for 24 days as shown in Fig. 3c. At the beginning, tumors were all suppressed by TMZ, but the tumors in shLuc group demonstrated faster growth than sh530 and sh532 groups after 15 days of treatment (Fig. 3d). Similarly, the tumor weight was lower in the knockdown groups, compared with the control group (Fig. 3e, f, ***p < 0.001). In this way, we demonstrated that the Skp2 knockdown sensitized glioma cells to TMZ both in vitro and in vivo.

### Skp2 involved in maintenance of stemness of glioma cells and suppression of senescence

**Fig. 4:**
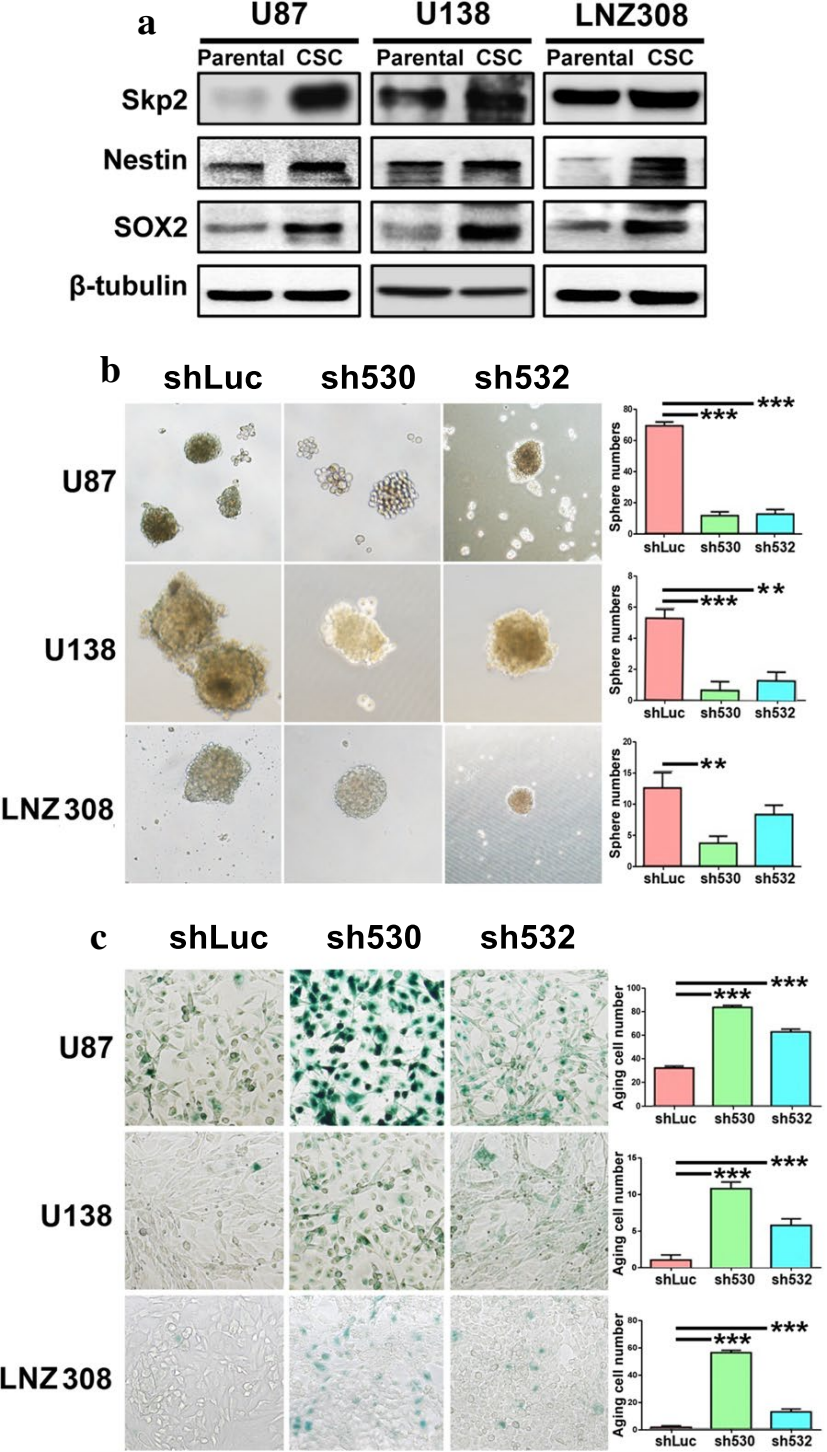
Downregulation of Skp2 reduced sphere formation ability of glioma stem-like cells and induced cellular senescence. **a** Skp2 protein levels were enhanced together with the glioma stem cell markers Nestin and Sox2 after enrichment with stem cell conditional medium in three glioma cell lines. **b** The sphere formation ability was reduced in all three cell lines upon Skp2 knockdown. **c** Cellular senescence was enhanced in the three cell lines upon Skp2 knockdown. **p < 0.01, ***p < 0.001

Glioma stem cells were considered responsible for the tumorigenicity as well as the resistance to chemotherapy and irradiation [20]. We previously reported that Skp2 is involved in self-renewal ability of hematopoietic stem cells and cancer stem cells in nasopharyngeal carcinoma [16, 17], therefore we speculated that Skp2 modulated glioma stem-like cells and thereafter regulated cell proliferation and drug sensitivity. Upon stem-like cell enrichment, the Skp2 level was significantly increased in U87 and U138 cells and slightly increased in LNZ308 cells accompanied by stem cell marker Nestin and Sox2 (Fig. 4a). The sphere formation ability was decreased dramatically upon Skp2-knockdown (**p < 0.01, ***p < 0.001, Fig. 4b). Repression of Skp2 induced senescence in glioma cells as reported in nasopharyngeal carcinoma (***p < 0.01, Fig. 4c) [21]. Taken together, we showed that Skp2 was involved in the stemness maintenance of cell and that the knockdown of Skp2 attenuated cell proliferation by decreasing stemness and increasing cell senescence.

### Small molecules targeting Skp2 sensitized glioma cells to TMZ

**Fig. 5:**
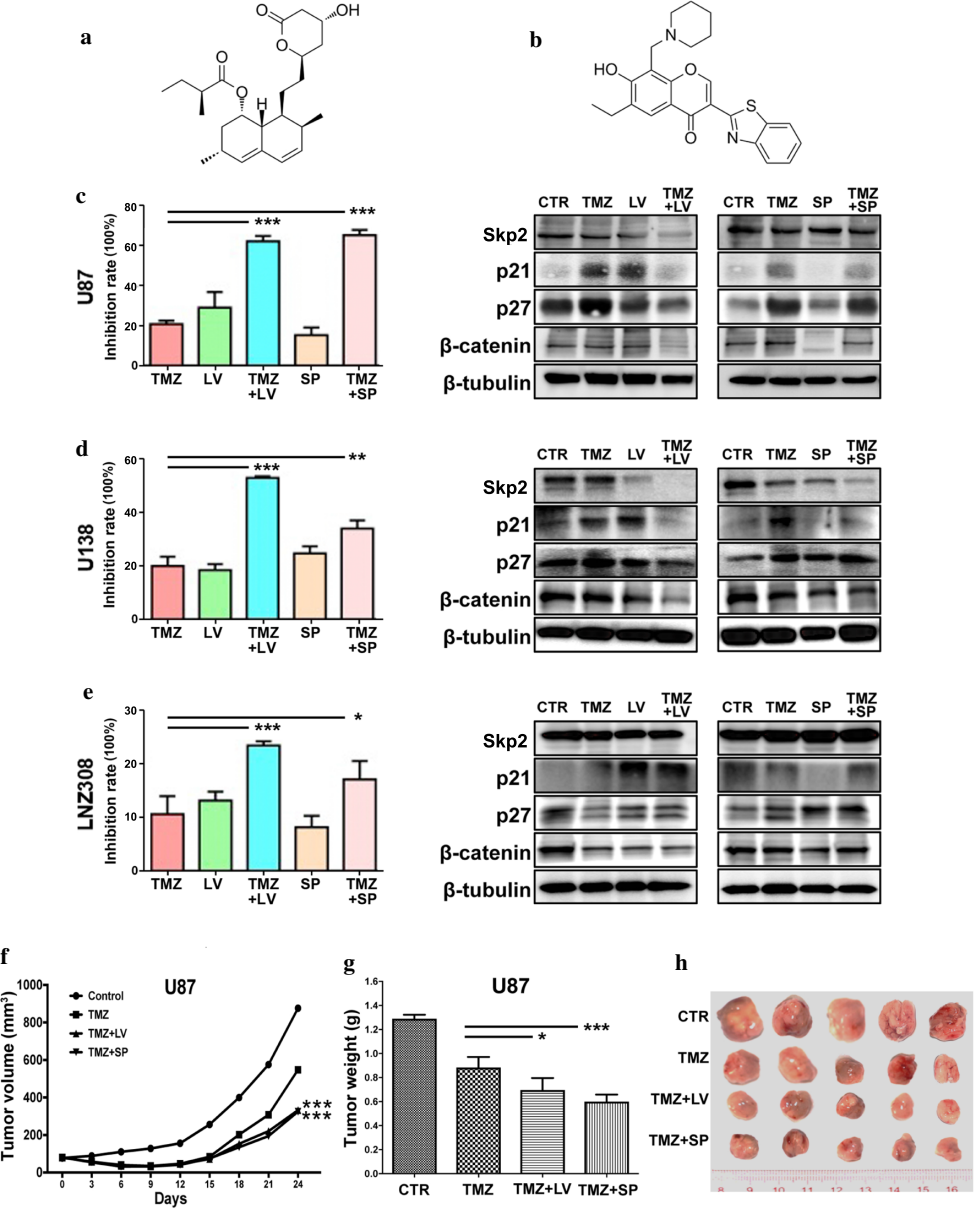
Combination therapy of TMZ and lovastatin or SZL-P1-41 was more effective than TMZ alone. **a**, **b** The chemical structures of lovastatin and SZL-P1-41. **c**–**e** The inhibitory effect was increased by the combined treatment of TMZ with lovastatin or SZL-P1-41, as shown in the left panels; in the right panels, the protein level of Skp2 and downstream targets p21^Cip1/Waf1^, p27^Kip1^ upon different treatments. **f** Tumor progression upon TMZ and lovastatin or SZL-P1-41 treatment. **g**, **h** Tumors and their corresponding weights from sacrificed mice. LV: lovastatin, SP: SZL-P1-41. *p < 0.05***p < 0.001

Targeting Skp2 might improve the therapeutic effects of glioma patients. The HMG-CoA reductase inhibitor lovastatin, widely used for lowering cholesterol and antineoplastic activities, induced degradation of Skp2 (Fig. 5a) [22]. Lovastatin may also arrest cells in the G1 phase of the cell cycle and therefore sensitize tumor cells to chemotherapeutic agents and ionizing radiation. In our case, lovastatin increased G1 phase cell number slightly in U138 and LNZ308 cells but not in U87 at 10 nM (Additional file 2: Figure S2), which might due to the relative lower concentration compared with other literatures (1 μM to 40 μM) [23, 24]. Similarly, lovastatin could not induce dramatic apoptosis in three cell lines at this concentration (Additional file 2: Figure S3). SZL-P1-41 prevents the assembly of Skp2-Skp1 complexes and inhibits Skp2 mediated ubiquitination of p27^Kip1^ and Akt, which therefore exhibits antitumor effects (Fig. 5b) [22, 25]. The IC50 of lovastatin and SZL-P1-41 were 14.4, 18.62 and 18.68 nmol/l and 20.2, 65.16 and 39.12 μmol/l in U87, U138, and LNZ308 cells, respectively. A concentration below the obtained IC50 was chosen (10 nmol/l for lovastatin, 20 μmol/l for SZL-P1-41) for combination treatment. The concentration of TMZ (500 μmol/l) was much lower than the clinically applied dose. The inhibition rates on three glioma cell lines were found to dramatically increase upon combined treatment (TMZ + LV or TMZ + SP) compared to TMZ treatment only (*p < 0.05, **p < 0.01, ***p < 0.001, left panels of Fig. 5c–e). At the same time, the level of Skp2 was reduced in all three cells upon lovastatin treatment and in U138 cells by SZL-P1-41 (right panels of Fig. 5c–e). Outcomes from mice models further validated the slow growth of the tumors, which corresponded to their low tumor weight upon combined treatment as compared to TMZ only (*p < 0.05, ***p < 0.001, Fig. 5f–h). Thus, Skp2 suppression enhanced cell sensitivity to TMZ both in vitro and in vivo.

## Discussion

Targeted therapy has the potential to inhibit the important molecules involved in tumor proliferation, invasion, metastasis, and drug resistance; preventing the progression and spread of cancer, paving the way for personalized medicine [26]. As an important E3 ligase, Skp2 recognizes substrates, processes them for ubiquitination-mediated degradation. The suppression of Skp2 led to the accumulation of its substrates like p21^Cip1/Waf1^ and p27^Kip1^, and was therefore found to be involved in the regulation of quiescence and self-renewal of hematopoietic stem cells [17, 27, 28]. Skp2 has been shown to be highly expressed in various types of tumors and play important roles in tumor development, especially in cell proliferation and drug sensitivity. An increase of Skp2 promoted cell growth, migration, and invasion in glioma cells [12]. In this study, Skp2 expression was found to be upregulated in glioma, and high-level of Skp2 predicted poor prognosis for patients with LGG. The Skp2 suppression delayed cell proliferation, sphere formation, and tumorigenesis, while the enhancement of Skp2 increased stem cell markers Nestin and Sox2 in glioma. Therefore, targeting Skp2 could be a promising strategy to improve therapeutic efficiency for glioma patients.

Several compounds that effectively suppress Skp2 expression have been tested in tumor cells. Selenonucleoside LJ-2618 was shown to be able to trigger G2/M cell cycle arrest in prostate cancer cells by promoting Skp2 degradation [11]. (−)-gochnatiolide B inhibited Skp2 in bladder cancer by attenuating cell proliferation [29]. Curcumin reduced cell viability and activated apoptosis by suppressing Skp2 in head and neck carcinoma [30]. Targeting Skp2 has also been attempted in glioma. Through repression of Skp2, physcion 8-*O*-β-glucopyranoside, escitalopram oxalate, curcumin, butylidenephthalid blocked cell cycle, inhibited proliferation, induced apoptosis, therefore attenuated the cell viability of glioma cells or in mice models [12, 31–34]. The small molecule SPZ-P1-41 was reported to be an effective inhibitor of the Skp2-Skp1 complex, which inhibited the recognition of substrates and further attenuated the degradation of substrates [22]. Our study provided the evidence that SPZ-P1-41 could successfully attenuate cell proliferation and sensitize cells to TMZ in both glioma cell and xenograft mice models.

Currently, drug repurposing has become of increasing interest and evolved as a promising field in cancer treatment [35, 36]. Lovastatin is a common cholesterol-lowering agent and used for primary prevention of coronary heart disease and to slow the progression of coronary atherosclerosis. It was reported that lovastatin induced Skp2 degradation by depleting geranylgeranyl pyrophosphate and therefore causing cell cycle arrest [37]. Our data confirmed that Skp2 was effectively decreased by lovastatin, and cell sensitivity to TMZ was improved significantly.

## Conclusion

In this study, we demonstrated that Skp2 increased with glioma grade and predicted a poor prognosis in diffuse glioma as well as LGG. We also demonstrated that the downregulation of Skp2 led to the retardation of cell proliferation, increased TMZ sensitivity, reduced the sphere formation ability of glioma stem-like cells, and enhanced cellular senescence. Furthermore, two small molecules (lovastatin and SZL-P1-41) could target Skp2 to improve TMZ efficiency in vitro and in xenograft mouse model in vivo. Our results therefore reveal the important role of Skp2 in glioma tumorigenesis, targeting Skp2 could potentially improve the therapeutic efficiency of glioma patients.

## Supplementary information

**Additional file 1: Table S1.** WHO classification and IDH mutation status of glioma cell lines applied in our study. **Table S2.** Clinical information of the LGG and GBM cohort included for our OS analysis from TCGA database.

**Additional file 2: Figure S1. a**–**c** The role of Skp2 expression in the OS of patients with IDH1^wt^ GBM, IDH^wt^ LGG and even IDH^mut^ LGG was analyzed. **d**, **e** The role of Skp2 expression in the OS of IDH^mut^ LGG patients with or without 1p19q co-deletion was analyzed. **Figure S2.** Cell cycle cascade rate were detected in three cell lines, U87, U138 and LNZ308, upon LV, TMZ or combined treatments. With regard to the strong effect of TMZ in glioma cells, we chose 10 nM as the combination concentration of lovastatin in our study. Lovastatin caused a little bit of G2/M arrest in U87 and LNZ308, but not in U138 cell. The G1 phase cell numbers were enhanced in U138 and LNZ308 cells upon lovastatin treatment. Lovastatin did not further induce G2/M arrest when combined with TMZ in U87 and U138. However, in LNZ308 cells, lovastatin promoted G2/M arrest upon TMZ treatments dramatically. (*p < 0.05, **p < 0.01, ***p < 0.001). **Figure S3.** Lovastatin barely induced cell apoptosis in U87 and U138 cells but promoted cell apoptosis in LNZ308 cells markedly at 10 nM. When combined with TMZ, lovastatin did not promote the apoptotic cell rates in U87, and even antagonized the apoptosis induction effect in U138 and LNZ308 cells. (*p < 0.05, ***p < 0.001).

## Data Availability

Not applicable.

## Abbreviations

Skp2: S-phase kinase-associated protein 2
TMZ: Temozolomide
GBM: Glioblastoma
LGG: Low-grade glioma
TNBC: Triple-negative breast cancer
EGF: Epidermal growth factor
bFGF: Basic fibroblast growth factor
OD: Observation density
IC50: Half-maximal inhibitory concentration
OS: Overall survival

## References

[1] Barth RF, Zhang Z, Liu T (2018). A realistic appraisal of boron neutron capture therapy as a cancer treatment modality. Cancer Commun.

[2] Ding L, Wang C, Cui Y, Han X, Zhou Y, Bai J, Li R (2018). S-phase kinase-associated protein 2 is involved in epithelial-mesenchymal transition in methotrexate-resistant osteosarcoma cells. Int J Oncol.

[3] Deng T, Yan G, Song X, Xie L, Zhou Y, Li J, Hu X, Li Z, Hu J, Zhang Y (2018). Deubiquitylation and stabilization of p21 by USP11 is critical for cell-cycle progression and DNA damage responses. Proc Natl Acad Sci USA.

[4] Jia T, Zhang L, Duan Y, Zhang M, Wang G, Zhang J, Zhao Z (2014). The differential susceptibilities of MCF-7 and MDA-MB-231 cells to the cytotoxic effects of curcumin are associated with the PI3K/Akt-SKP2-Cip/Kips pathway. Cancer Cell Int.

[5] Meyer SE, Muench DE, Rogers AM, Newkold TJ, Orr E, O’Brien E, Perentesis JP, Doench JG, Lal A, Morris PJ (2018). miR-196b target screen reveals mechanisms maintaining leukemia stemness with therapeutic potential. J Exp Med.

[6] Wei X, Li X, Yan W, Zhang X, Sun Y, Zhang F (2018). SKP2 promotes hepatocellular carcinoma progression through nuclear AMPK-SKP2-CARM1 signaling transcriptionally regulating nutrient-deprived autophagy induction. Cell Physiol Biochem.

[7] Wang X, Zhang S, Zhang T, Shan J (2019). Prognostic values of F-box members in breast cancer: an online database analysis and literature review. Biosci Rep..

[8] Huo J, Chen X, Zhang H, Hu Y, Jiang Y, Liu S, Zhang X (2018). Bcl-3 promotes proliferation and chemosensitivity in BL1 subtype of TNBC cells. Acta Biochim Biophys Sin.

[9] Zhong K, Yang F, Han Q, Chen J, Wang J (2018). Skp2 expression has different clinicopathological and prognostic implications in lung adenocarcinoma and squamous cell carcinoma. Oncol Lett.

[10] Zhang Y, Zvi YS, Batko B, Zaphiros N, O’Donnell EF, Wang J, Sato K, Yang R, Geller DS, Koirala P (2018). Down-regulation of Skp2 expression inhibits invasion and lung metastasis in osteosarcoma. Sci Rep.

[11] Byun WS, Jin M, Yu J, Kim WK, Song J, Chung HJ, Jeong LS, Lee SK (2018). A novel selenonucleoside suppresses tumor growth by targeting Skp2 degradation in paclitaxel-resistant prostate cancer. Biochem Pharmacol.

[12] Wang L, Ye X, Cai X, Su J, Ma R, Yin X, Zhou X, Li H, Wang Z (2015). Curcumin suppresses cell growth and invasion and induces apoptosis by down-regulation of Skp2 pathway in glioma cells. Oncotarget.

[13] Tang Z, Li C, Kang B, Gao G, Li C, Zhang Z (2017). GEPIA: a web server for cancer and normal gene expression profiling and interactive analyses. Nucleic Acids Res.

[14] Tang Z, Kang B, Li C, Chen T, Zhang Z (2019). GEPIA2: an enhanced web server for large-scale expression profiling and interactive analysis. Nucleic Acids Res.

[15] Chan CH, Lee SW, Li CF, Wang J, Yang WL, Wu CY, Wu J, Nakayama KI, Kang HY, Huang HY (2010). Deciphering the transcriptional complex critical for RhoA gene expression and cancer metastasis. Nat Cell Biol.

[16] Wang J, Huang Y, Guan Z, Zhang JL, Su HK, Zhang W, Yue CF, Yan M, Guan S, Liu QQ (2014). E3-ligase Skp2 predicts poor prognosis and maintains cancer stem cell pool in nasopharyngeal carcinoma. Oncotarget.

[17] Wang J, Han F, Wu J, Lee SW, Chan CH, Wu CY, Yang WL, Gao Y, Zhang X, Jeong YS (2011). The role of Skp2 in hematopoietic stem cell quiescence, pool size, and self-renewal. Blood.

[18] Feng HB, Wang J, Jiang HR, Mei X, Zhao YY, Chen FR, Qu Y, Sai K, Guo CC, Yang QY (2017). Beta-elemene selectively inhibits the proliferation of glioma stem-like cells through the downregulation of Notch1. Stem Cells Transl Med.

[19] Louis DN, Perry A, Reifenberger G, von Deimling A, Figarella-Branger D, Cavenee WK, Ohgaki H, Wiestler OD, Kleihues P, Ellison DW (2016). The 2016 World Health Organization classification of tumors of the central nervous system: a summary. Acta Neuropathol.

[20] Yawata T, Higashi Y, Kawanishi Y, Nakajo T, Fukui N, Fukuda H, Ueba T (2019). CD146 is highly expressed in glioma stem cells and acts as a cell cycle regulator. J Neurooncol.

[21] Tsai YC, Kuo PL, Kuo MC, Hung WW, Wu LY, Chang WA, Wu PH, Lee SC, Chen HC, Hsu YL (2018). The interaction of miR-378i-Skp2 regulates cell senescence in diabetic nephropathy. J Clin Med.

[22] Chan CH, Morrow JK, Li CF, Gao Y, Jin G, Moten A, Stagg LJ, Ladbury JE, Cai Z, Xu D (2013). Pharmacological inactivation of Skp2 SCF ubiquitin ligase restricts cancer stem cell traits and cancer progression. Cell.

[23] Macaulay RJ, Wang W, Dimitroulakos J, Becker LE, Yeger H (1999). Lovastatin-induced apoptosis of human medulloblastoma cell lines in vitro. J Neurooncol.

[24] Bar EE, Chaudhry A, Farah MH, Eberhart CG (2007). Hedgehog signaling promotes medulloblastoma survival via Bc/II. Am J Pathol.

[25] Mikamo M, Kitagawa K, Sakai S, Uchida C, Ohhata T, Nishimoto K, Niida H, Suzuki S, Nakayama KI, Inui N (2018). Inhibiting Skp2 e3 ligase suppresses bleomycin-induced pulmonary fibrosis. Int J Mol Sci.

[26] Marchio C, Balmativola D, Castiglione R, Annaratone L, Sapino A (2017). Predictive diagnostic pathology in the target therapy era in breast cancer. Curr Drug Targets.

[27] Hu L, Ibrahim S, Liu C, Skaar J, Pagano M, Karpatkin S (2009). Thrombin induces tumor cell cycle activation and spontaneous growth by down-regulation of p27Kip1, in association with the up-regulation of Skp2 and MiR-222. Cancer Res.

[28] Lee SH, McCormick F (2005). Downregulation of Skp2 and p27/Kip1 synergistically induces apoptosis in T98G glioblastoma cells. J Mol Med.

[29] Chen Y, Li W, Zeng Z, Tang Y (2018). (−)-Gochnatiolide B, synthesized from dehydrocostuslactone, exhibits potent anti-bladder cancer activity in vitro and in vivo. Sci Rep.

[30] Khan AQ, Siveen KS, Prabhu KS, Kuttikrishnan S, Akhtar S, Shaar A, Raza A, Mraiche F, Dermime S, Uddin S (2018). Curcumin-mediated degradation of S-phase kinase protein 2 induces cytotoxic effects in human papillomavirus-positive and negative squamous carcinoma cells. Front Oncol.

[31] Li W, Li F, Zhu Y, Song D (2017). Physcion 8-*O*-beta-glucopyranosideregulates cell cycle, apoptosis, and invasion in glioblastoma cells through modulating Skp2. Biomed Pharmacother.

[32] Chen VC, Hsieh YH, Chen LJ, Hsu TC, Tzang BS (2018). Escitalopram oxalate induces apoptosis in U-87MG cells and autophagy in GBM8401 cells. J Cell Mol Med.

[33] Ouyang J, Xu H, Li M, Dai X, Fu F, Zhang X, Lan Q (2018). Paeoniflorin exerts antitumor effects by inactivating S phase kinase-associated protein 2 in glioma cells. Oncol Rep.

[34] Huang MH, Lin SZ, Lin PC, Chiou TW, Harn YW, Ho LI, Chan TM, Chou CW, Chuang CH, Su HL (2014). Brain tumor senescence might be mediated by downregulation of S-phase kinase-associated protein 2 via butylidenephthalide leading to decreased cell viability. Tumour Biol.

[35] Seliger C, Hau P (2018). Drug repurposing of metabolic agents in malignant glioma. Int J Mol Sci.

[36] Cancer C (2018). The 150 most important questions in cancer research and clinical oncology series: questions 94–101: Edited by Cancer Communications. Cancer Commun.

[37] Vosper J, Masuccio A, Kullmann M, Ploner C, Geley S, Hengst L (2015). Statin-induced depletion of geranylgeranyl pyrophosphate inhibits cell proliferation by a novel pathway of Skp2 degradation. Oncotarget.

