# Supplementary material for: Skp2 modulates proliferation, senescence and tumorigenesis of glioma
Source: Cancer Cell Int. 2020 Mar 6;20:71. doi: 10.1186/s12935-020-1144-z (PMC7059397; doi:10.1186/s12935-020-1144-z)
Supplement: Supplementary file 1 — Additional file 1: Table S1. WHO classification and IDH mutation status of glioma cell lines applied in our study. Table S2. Clinical information of the LGG and GBM cohort included for our OS analysis from TCGA database. [file 12935_2020_1144_MOESM1_ESM.docx]

***Table S1. WHO classification and IDH mutation status of glioma cell lines applied in our study.***

| ***Cell line*** | ***Age/Gender*** | ***Tumor stage*** | ***IDH1*** |
| --- | --- | --- | --- |
| U-87MG | 44 yr / Female | Grade IV | Wide type |
| U-118MG | 50 yr / Male | Grade IV | Wide type |
| U-138MG | 47 yr / Male | Grade IV | Wide type |
| U-343MG | 60 yr / Male | Grade IV | Wide type |
| U-373MG | 50 yr / Male | Grade IV | Wide type |
| LNZ308 | 60 yr / Male | Grade IV | Wide type |
| A172 | 53 yr / Male | Grade IV | Wide type |

|  | All cases | LGG | | GBM | |
| --- | --- | --- | --- | --- | --- |
| Age |  |  | |  | |
| <49 | 326 | 298 | | 28 | |
| ≥49 | 335 | 212 | | 123 | |
| Unknown | 0 | 0 | | 0 | |
| Total | 661 | 510 | | 151 | |
| Gender |  |  | |  | |
| Male | 352 | 254 | | 98 | |
| Female | 252 | 199 | | 53 | |
| Unknown | 57 | 57 | | 0 | |
| Total | 661 | 510 | | 151 | |
| WHO Grade |  |  | |  | |
|  |  | I | 0 | IV | 151 |
|  |  | II | 226 |  | |
|  |  | III | 240 |  | |
|  |  | Unknown | 44* |  | |
|  |  | Total | 510 | Total | 151 |
| IDH1 |  |  | |  | |
| Mutant | 423 | 1p19q Co-deletion | 168 | 0 | |
|  |  | Non-co-deletion | 245 | 8 | |
|  |  | Unknown | 0 | 2 | |
| WT | 231 | 94 | | 137 | |
| Unknown | 7 | 3 | | 4 | |
| Total | 661 | 510 | | 151 | |

***Table S2. Clinical information of the LGG and GBM cohort included for our overall survival analysis from TCGA database.***

*: the exact grade of the tumor was unknown, but the tumor diagnosed as Astrocytoma or Oligoastrocytoma, which were all considered to be low grade glioma (LGG). WT, wild type.
