# Supplementary material for: Skp2 modulates proliferation, senescence and tumorigenesis of glioma
Source: Cancer Cell Int. 2020 Mar 6;20:71. doi: 10.1186/s12935-020-1144-z (PMC7059397; doi:10.1186/s12935-020-1144-z)
Supplement: Supplementary file 2 — Additional file 2: Figure S1. a–c The role of Skp2 expression in the OS of patients with IDH1wt GBM, IDHwt LGG and even IDHmut LGG was analyzed. d, e The role of Skp2 expression in the OS of IDHmut LGG patients with or without 1p19q co-deletion was analyzed. Figure S2. Cell cycle cascade rate were detected in three cell lines, U87, U138 and LNZ308, upon LV, TMZ or combined treatments. With regard to the strong effect of TMZ in glioma cells, we chose 10 nM as the combination concentration of lovastatin in our study. Lovastatin caused a little bit of G2/M arrest in U87 and LNZ308, but not in U138 cell. The G1 phase cell numbers were enhanced in U138 and LNZ308 cells upon lovastatin treatment. Lovastatin did not further induce G2/M arrest when combined with TMZ in U87 and U138. However, in LNZ308 cells, lovastatin promoted G2/M arrest upon TMZ treatments dramatically. (*p < 0.05, **p < 0.01, ***p < 0.001). Figure S3. Lovastatin barely induced cell apoptosis in U87 and U138 cells but promoted cell apoptosis in LNZ308 cells markedly at 10 nM. When combined with TMZ, lovastatin did not promote the apoptotic cell rates in U87, and even antagonized the apoptosis induction effect in U138 and LNZ308 cells. (*p < 0.05, ***p < 0.001). [file 12935_2020_1144_MOESM2_ESM.doc]

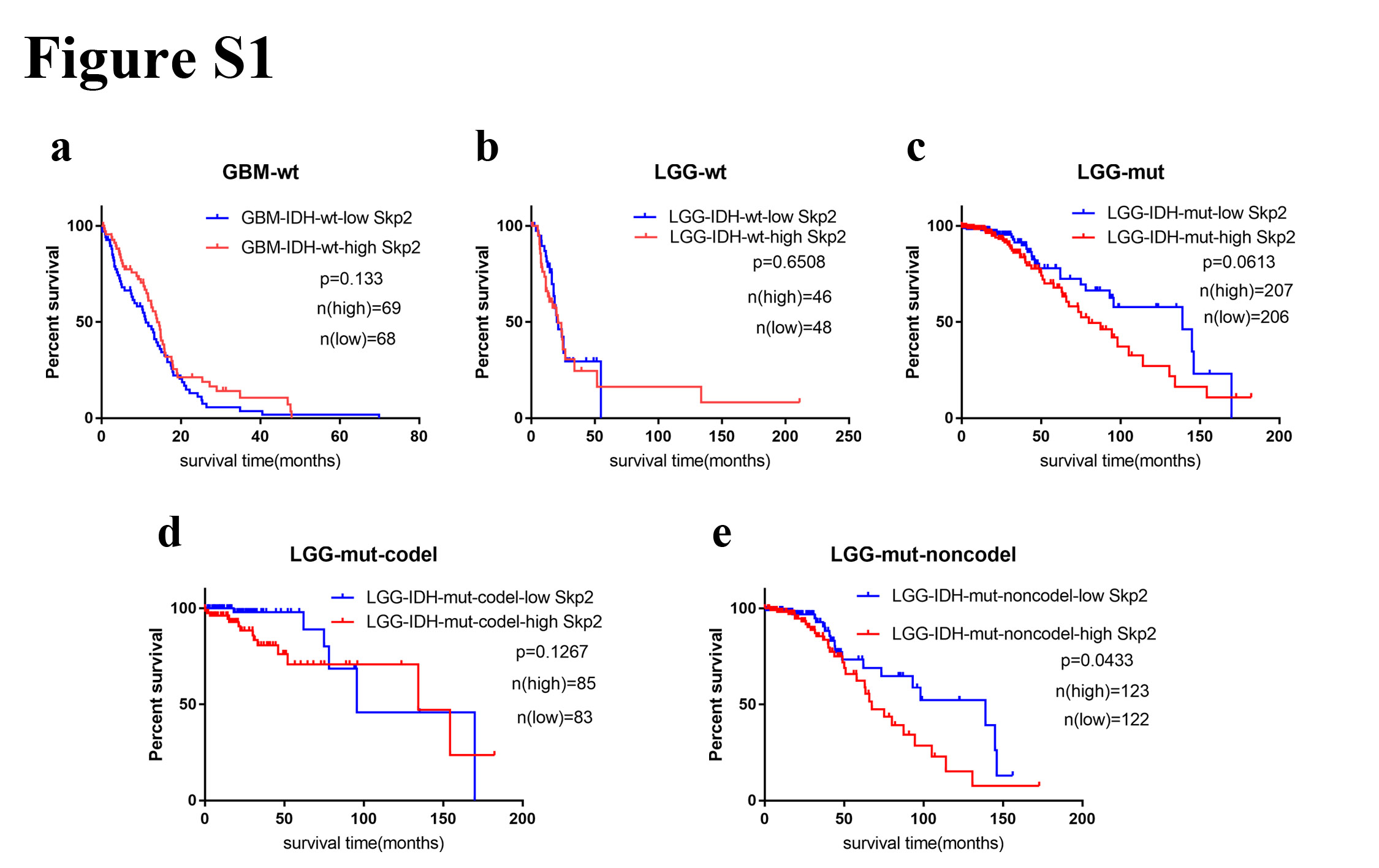


- **Figure legends:** Figure S1: **a-c** The role of Skp2 expression in the OS of patients with IDH1^wt^ GBM, IDH^wt^ LGG and even IDH^mut^ LGG was analyzed. **d-e** The role of Skp2 expression in the OS of IDH^mut^ LGG patients with or without 1p19q co-deletion was analyzed.


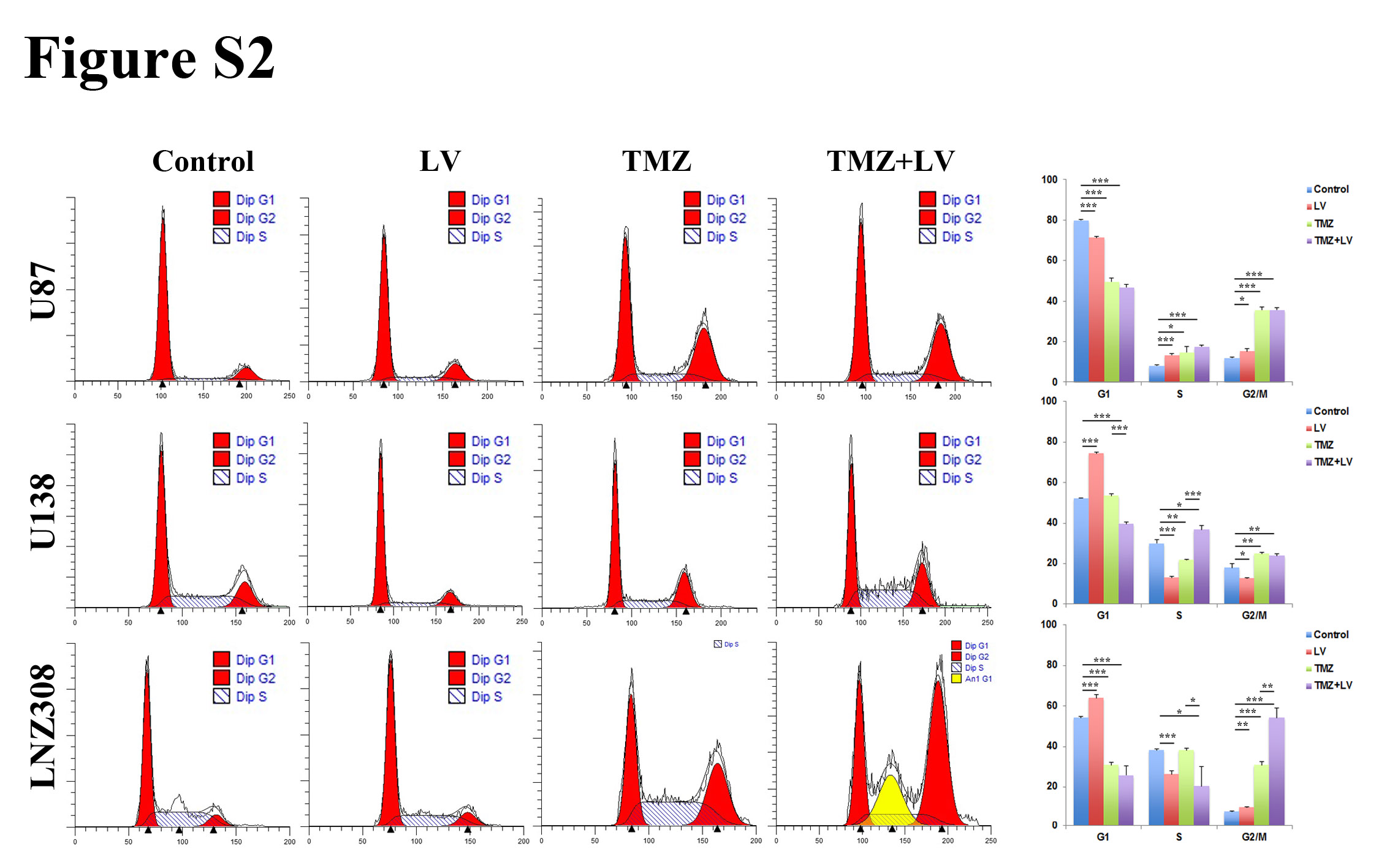


- **Figure legends:** Figure S2: Cell cycle cascade rate were detected in three cell lines, U87, U138 and LNZ308, upon LV, TMZ or combined treatments. With regard to the strong effect of TMZ in glioma cells, we chose 10nM as the combination concentration of lovastatin in our study.   Lovastatin caused a little bit of G2/M arrest in U87 and LNZ308, but not in U138 cell. The G1 phase cell numbers were enhanced in U138 and LNZ308 cells upon lovastatin treatment. Lovastatin did not further induce G2/M arrest when combined with TMZ in U87 and U138. However, in LNZ308 cells, lovastatin promoted G2/M arrest upon TMZ treatments dramatically. (*: p<0.05, ***:p<0.001).


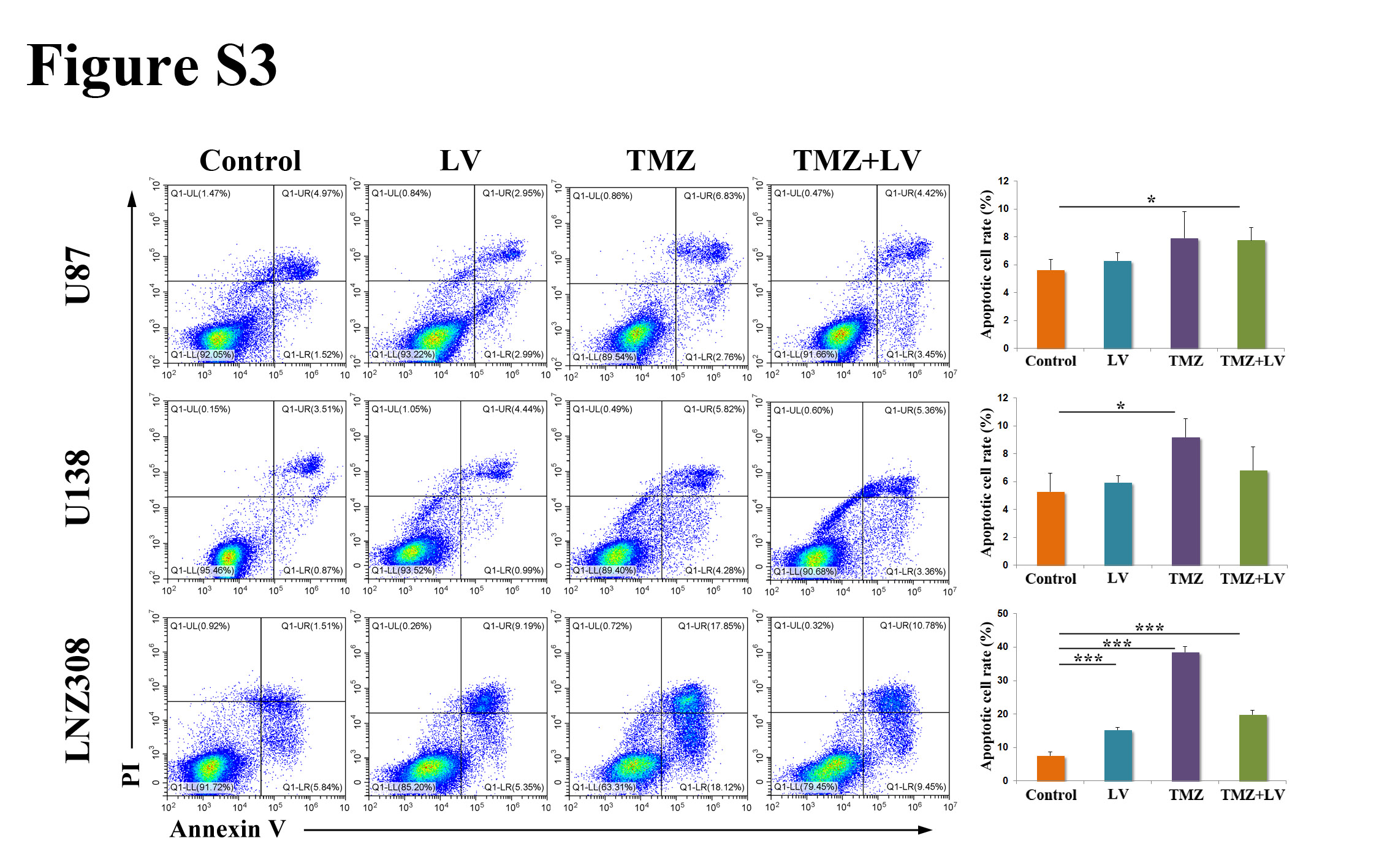


- **Figure legends:** Figure S3: Lovastatin barely induced cell apoptosis in U87 and U138 cells but promoted cell apoptosis in LNZ308 cells markedly at 10nM. When combined with TMZ, lovastatin did not promote the apoptotic cell rates in U87, and even antagonized the apoptosis induction effect in U138 and LNZ308 cells. (*: p<0.05, **: p<0.01, ***:p<0.001).
